# Lymphoma-associated hemophagocytic lymphohistiocytosis

**DOI:** 10.1016/j.htct.2025.106087

**Published:** 2025-11-18

**Authors:** Thomás de Souza Patto Marcondes, Carlos Sérgio Chiattone, Rafael Dezen Gaiolla

**Affiliations:** a Universidade Estadual Paulista (Unesp), Faculdade de Medicina de Botucatu, Departamento de Clínica Médica Geral, Hematologia, Botucatu, SP, Brazil; b Hospital das Clínicas da Faculdade de Medicina de Botucatu, Botucatu, SP, Brazil; c Faculdade de Ciências Médicas da Santa Casa de Misericórdia de São Paulo, São Paulo, SP, Brazil; d Hospital Samaritano Higienópolis, São Paulo, SP, Brazil

**Keywords:** Hemophagocytic lymphohistiocytosis, Lymphoma

## Abstract

Hemophagocytic lymphohistiocytosis is a severe, rare condition characterized by excessive immune activation, leading to significant morbidity and mortality. Lymphoma is the most common trigger for malignancy-related hemophagocytic lymphohistiocytosis in adults, with large B-cell non-Hodgkin, T- and NK-cell lymphomas being the most diagnosed. Hodgkin lymphoma is less frequently observed. Lymphoma-associated hemophagocytic lymphohistiocytosis poses diagnostic and therapeutic challenges due to its complex pathogenesis and heterogeneous presentation. Treatment aims to control the overactive immune system, identify and treat modifying factors, optimize clinical support, and treat the underlying lymphoma. Early etoposide (Etoposide) combined with dexamethasone for immunomodulation results in rapid control of hyperinflammation and clinical improvement. It has increasingly been adopted as a standard initial approach followed by lymphoma-specific treatment. However, the outcomes for patients with lymphoma-associated hemophagocytic lymphohistiocytosis remain poor, especially for patients with T- and NK-cell lymphomas. In relapsed or refractory cases, emerging therapies have been explored, with ruxolitinib showing the most promising results. This paper reviews current understanding of the epidemiology, pathogenesis, clinical features, diagnosis, and treatment of lymphoma-associated hemophagocytic lymphohistiocytosis in adults and proposes an appropriate treatment protocol based on the most recent data from the literature.

## Introduction

Hemophagocytic lymphohistiocytosis (HLH) is a spectrum of conditions characterized by intense, pathological immune activation with clinical manifestations such as extreme inflammation, hemophagocytosis, and end-organ damage. HLH is rare and is most frequently diagnosed in children, but 30–40 % of cases are observed in adults [1]. HLH is classified as an H-group histiocytic disorder and is historically divided into primary or secondary HLH.

Primary HLH is predominantly observed in children whose genetic defects lead to inflammasome impairment or disturbance, particularly of cytotoxic T-cells and natural killer (NK) cells. Secondary HLH is mainly observed in adults, with immune activation being caused by external triggers, including persistent infection, autoimmune conditions, and malignancy [2,3]. However, recent findings suggest that 40 % of adult patients have genetic abnormalities that mainly affect the perforin cytosolic pathway. Based on its pathophysiology, HLH is currently defined as a clinical spectrum of conditions with a predisposition to hyperinflammation with predisposing factors that include genetic defects, immune impairment, and acute triggers. [4,5]

Malignancy is the leading cause of HLH in adults, diagnosed in up to 50 % of cases. Hematological neoplasms and lymphomas are the most common triggers of malignancy-associated HLH (M-HLH) in adults [1,6]. This paper reviews the pathogenesis and diagnosis of lymphoma-associated HLH (L-HLH) in adults and proposes an appropriate treatment protocol based on the most recent data from the literature.

## Epidemiology

HLH is a rare and often underdiagnosed disease. Therefore, the incidence and prevalence in adults remain unclear [7,8]. Available studies based on small cohorts estimate an annual incidence lower than 1 case per 100,000 people per year [1,9]. The median age is approximately 50 years, with a clear predominance of females (7:1).

In assessing the epidemiology of M-HLH, a Swedish population-based study analyzed all patients with neoplastic and histiocytic disorders registered in the national database and reported an incidence of 0.21 cases per 100,000 inhabitants, with a male predominance. Lymphoma was the most common malignancy diagnosed in M-HLH [10].

In hematologic malignancies, approximately 1 % develop HLH, detected either at the time of initial cancer diagnosis or during treatment [9]. Lymphomas are the most common triggers, accounting for 45–50 % of patients. Of these, T- and NK-cell lymphomas/leukemias are diagnosed in approximately 35 % of cases; large B-cell non-Hodgkin lymphomas (NHL) in 32 %; and Hodgkin lymphoma in 6 % [1]. Uncommon subtypes of B- (intravascular B-cell lymphoma) and T-cell (nasal NK/T-cell, angioimmunoblastic T-cell, gamma/delta T-cell, and subcutaneous panniculitis-like T-cell lymphoma) NHL must also be considered because they account for 20 % of adults with L-HLH [1,11]*.*

A key epidemiological finding is the geographic variability in the incidence of different subtypes of lymphomas associated with HLH. T-cell and NK-cell lymphomas prevail in Eastern countries, such as China and Japan. In contrast, Western countries tend towards an equal distribution of B- and T-cell lymphomas [1]. The reasons for this geographic variability are unclear, but they may be related to Epstein-Barr virus infection, which is more prevalent in eastern countries, and genetic differences between these populations [12,13]. A recent study evaluated 173 patients with intravascular B-cell lymphoma, of whom 50 were from Western countries and 123 from Eastern countries. None of the patients from Western countries presented with L-HLH in this series. Conversely, 45 patients from eastern countries were diagnosed with L-HLH at some point. These findings suggest a genetic predisposition to L-HLH [14].

HLH can also be triggered by lymphoma treatment (known as treatment-related HLH), including chemotherapy, hematopoietic stem cell transplantation (HSCT), and, more recently, checkpoint inhibitors and targeted cell therapy, such as chimeric antigen receptor (CAR) T-cell and bispecific antibody therapy. The incidence is highly variable, reaching 30 % in some series. Moreover, the diagnosis is challenging due to potential confounding factors, such as lymphoma activity and secondary infections [15,16].

## Lymphoma-associated hemophagocytic lymphohistiocytosis pathogenesis

Under normal physiology, the immune response is an orchestrated process involving interactions between immune cells and proteins, such as granulocytes, lymphocytes, macrophages, immunoglobulins, cytokines, and complement molecules. Each condition that increases the inflammatory response is counteracted by a process that avoids excessive and dangerous immune stimulation, which could lead to tissue destruction. In HLH, this process is disrupted, particularly the autoregulatory mechanisms, resulting in excessive and persistent inflammation and organ damage [3,17].

Primary (or familial) HLH is considered a model for understanding the pathophysiology of this condition. In these patients, recessive mutations in genes involved in T- and NK-cell cytotoxicity cause defective perforin and granzyme secretion, impairing the ability to clear the antigenic stimulus and downregulate the inflammatory response [4,15,18]. In turn, the pathophysiology of secondary (or acquired) HLH is not entirely understood and is likely to be multifactorial. One of the main mechanisms proposed so far is the constant presence of an antigenic stimulus, resulting in CD8^+^ *T*- and NK-cell hyperactivation and, consequently, excessive secretion of proinflammatory cytokines and increased macrophage activation. Furthermore, hereditary genetic alterations associated with immune response defects previously considered only in primary HLH have also been identified in secondary HLH and may contribute to its pathogenesis [4,8,18, 19, 20, 21].

In M-HLH, particularly L-HLH, the immune dysfunction is also intrinsic to neoplasia or triggered by the different treatment modalities, leading to immune activation and loss of immune inhibitory function [19].

Finally, increased predisposition to bacterial and viral infection at diagnosis and during lymphoma treatment is a significant risk factor for L-HLH. Among the primary pathogens, Epstein-Barr virus stands out because chronic infection with this virus is directly related to the development of some types of lymphomas, such as Burkitt and T-cell lymphomas, and plays a key role in regulating the immune response to neoplasias [13,22].

## Clinical presentation and diagnosis

L-HLH has a challenging diagnosis; it may be detected at diagnosis, or during relapse or treatment of lymphoma. L-HLH is often triggered by an uncommon lymphoma histological subtype with an atypical clinical presentation [11]. Moreover, some signs and symptoms of L-HLH are not specific to this disease and may be explained by lymphoma or because of treatment.

Clinically, L-HLH presents as an acute or subacute febrile condition associated with multiple organ dysfunction. Fever is present in >90 % of patients, usually associated with cytopenia in at least two lineages [6,23]. Other commonly observed findings in L-HLH include markedly elevated lactate dehydrogenase (LDH), ferritin, and soluble CD25 (sCD25), which are almost universally present [23]. Organic involvement may manifest as hepatocellular injury, which may or may not be associated with impaired liver function, hepatosplenomegaly, neurological changes, skin lesions, bleeding, or acute respiratory distress syndrome [1,7,11,24,25]. Moreover, most patients with L-HLH are diagnosed in advanced Ann-Arbor stages and frequently have lymphoma infiltration in the bone marrow and other extranodal sites, like the liver and skin [23].

Different criteria grouped into scoring protocols have been proposed for a more accurate diagnosis of HLH. These are also used for the diagnosis of L-HLH. The oldest and most widely used are the revised HLH-2004 diagnostic criteria from the Histiocyte Society, which are based on eight clinical, laboratory, and cytopathological criteria (Table 1). Diagnostic confirmation requires meeting at least five of the eight criteria [26].

**Table 1:** HLH-2004 diagnostic guidelines for hemophagocytic lymphohistiocytosis.

At least five of the following criteria: Fever Splenomegaly Cytopenias (affecting at least two lineages): Hemoglobin <9 g/dL Platelets <100.000/mm^3^ Neutrophils <1.000/mm^3^ Hypertriglyceridemia and/or hypofibrinogenemia Fasting triglycerides ≥265 mg/dL fibrinogen <150 mg/dL Hemophagocytosis in bone marrow, spleen, liver or lymph nodes Ferritin >500 mg/dL Reduced or absent NK-cell function Increased serum levels of CD25 (soluble IL-2 receptor) ≥2400 U/mL

These scoring protocols were initially developed for children with familial HLH. While they capture the most frequently observed alterations in adult HLH, their sensitivity and specificity have not been prospectively validated in adults. Additionally, HLH-2004 uses nonspecific parameters that may overlap with other inflammatory conditions or manifestations of lymphoma and overlooks alterations frequently observed in HLH, such as increased transaminase, lactic dehydrogenase, D-dimer, and C-reactive protein levels and neurological manifestations. Therefore, while HLH-2004 should be applied in clinical practice, the findings should be interpreted with caution, particularly where there is high suspicion of HLH without fully meeting the criteria [11,20,27].

Alternative guidelines have been proposed to improve HLH diagnosis in adults. Based on data from a retrospective cohort, the HScore was developed to define and predict the likelihood of adult HLH. After weighing the significant clinical and laboratory parameters, the authors identified an optimal cutoff of 169 points with 93 % sensitivity and 86 % specificity (Table 2). Notably, 44 % of the study cohort were patients with cancer [28]. In a retrospective analysis, the accuracy of the HScore was directly compared with that of HLH-2004, with the former outperforming the latter, reaching 90 % sensitivity and 79 % specificity for adults at the initial presentation. However, the values were similar when clinical status was worse. Moreover, the optimal cutoff can be affected by the trigger and pattern of the individual inflammatory response. These factors may impact the sensitivity and specificity of the HScore in different cohorts [29,30].

**Table 2:** HScore.

Parameter	Points for scoring
1. Fever ( °C)	0 (<38.4), 33 (38.4 −39.4) or 49 (>39.4)
2. Cytopenia	0 (1 lineage), 24 (2), 34 (3)
3. Organomegaly	0 (no), 23 (hepatomegaly or splenomegaly) or 38 (hepatomegaly and splenomegaly)
4. Ferritin (ng/mL)	0 (<2000), 35 (2000–6000) or 50 (>6000)
5. Fibrinogen (mg/dL)	0 (>250) or 30 (≤250)
6. Triglycerides (mg/dL)	0 (<150), 44 (150–400), 64 (>400)
7. Aspartate aminotransferase	0 (<30) or 19 (≥30)
8. Hemophagocytosis on biopsy	0 (no) or 35 (yes)
9. Known underlying immunosuppression	0 (no) or 18 (yes)

In a retrospective database analysis that included only patients with complete documentation of cancer, HLH-2004 criteria were compared with extended diagnostic criteria comprising 18 variables. The authors of this study, conducted at the MD Anderson Cancer Center, University of TX, reported that among patients with suspected HLH, only 21 % met the standard HLH-2004 diagnostic criteria, whereas 57 % met the extended diagnostic criteria. No significant difference in outcome (overall survival [OS]) was found between the 13 patients who met the HLH-2004 criteria and the 20 patients who did not meet the HLH-2004 criteria but met the extended 18-point HLH criteria, suggesting that these patients are likely to have had a more aggressive systemic process. Case in point, the OS was significantly improved among the 26 patients with hemophagocytosis or lymphohistiocytosis on pathological examination but failed to meet either HLH-2004 or expanded HLH criteria [31].

More recently, to improve and simplify the specific diagnosis of M-HLH, the roles of sCD25 and ferritin as potential diagnostic biomarkers were studied in a multicenter retrospective cohort of 225 patients [32]. Patients with and without HLH were included, all of whom met the HLH-2004 criteria. Among different HLH diagnostic parameters, the optimized HLH inflammatory (OHI) index, a composite score defined by the simultaneous elevation of sCD25 (>3900 U/mL) and ferritin (>1000 ng/mL), provides an accurate diagnosis, yielding a prognostic tool with 84 % sensitivity and 81 % specificity. OHI highly predicted mortality across hematologic malignancies, but this combined index still requires validation in larger cohorts. The kinetics of sCD25 have also been explored as a predictor of survival. Verkamp et al. demonstrated that the failure to improve sCD25 from baseline strongly predicted survival in children and young adults treated with etoposide (Etoposide)-based therapy. Additionally, the combination of sCD25 with other biomarkers such as platelet count, absolute lymphocyte count, and blood urea nitrogen also predicted mortality, suggesting a potential role in the early identification of high-risk patients [33].

Despite these tools, diagnosis of L-HLH remains challenging because symptoms are usually nonspecific, and lymphoma can be difficult to detect on physical examination. For an early diagnosis, physicians must identify hyperinflammation based on clinical and laboratory findings, including rapid clinical deterioration, persistent fever, a high ferritin level, and cytopenia. Atypical clinical presentations and less frequent histological subtypes should also be considered (Table 3). Ancillary methods, such as positron emission tomography-computed tomography-guided tissue biopsy, bone marrow biopsy, and flow cytometry, may facilitate the diagnosis. Another relevant issue is the limited access to specific diagnostic tests required by the HLH-2004 criteria, such as sCD25 levels and NK cell activity, particularly in resource-limited settings. Without a gold standard scoring protocol for diagnosing L-HLH, new and accurate biomarkers must be urgently developed to provide rapid confirmation of diagnosis and timely initiation of treatment towards improving outcomes for patients with L-HLH.

**Table 3:** The most common lymphoma subtypes associated with lymphoma-associated hemophagocytic lymphohistiocytosis.

**T-cell and NK-cell lymphomas**	**B-cell lymphomas**
NK/T-cell lymphoma, aggressive NK cell leukemia Peripheral T-cell lymphoma Anaplastic large cell lymphoma; Angioimmunoblastic T-cell lymphoma Panniculitis-like T-cell NHL Gamma-delta T-cell lymphoma	DLBCL Intravascular B-cell lymphoma Indolent lymphomas (FL, MZL) Burkitt lymphoma Hodgkin lymphoma

NK: Natural killer; DLBCL: diffuse large B-cell lymphoma; FL: follicular lymphoma; MZL: marginal zone lymphoma.

## Treatment

**Figure 1:**
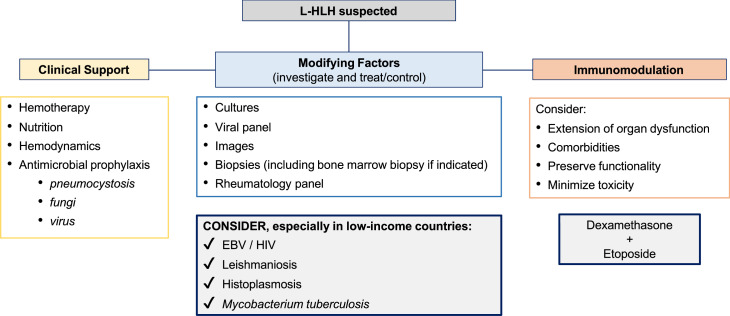
General Principles of Lymphoma-associated hemophagocytic lymphohistiocytosis (L-HLH) Treatment. The mainstay of L-HLH treatment is to control the overactive immune system, identify and treat modifying factors, optimize clinical support, and treat lymphoma. EBV: Epstein–Barr virus.

L-HLH is a life-threatening condition with challenging treatment. Most patients undergoing treatment are aged and very ill, with chemotherapy and immunosuppressive therapy increasing the risk of complications. The condition frequently requires rapid intervention, so measures to improve health status before initiating treatment cannot be implemented. L-HLH treatment aims to control the overactive immune system, identify and treat modifying factors, optimize clinical support, and treat lymphoma [5,34,35] (Figure 1). However, due to the lack of prospective, randomized, or controlled clinical trials, there is no consensus on whether an HLH, malignancy-directed, or combined approach should be adopted first. Most available data derive from very small retrospective series and case reports, with potential selection bias. The strength of the recommendations is usually based on expert opinions [11,20,36].

In general, the prognosis of patients with L-HLH is poor. Different series in the literature show heterogeneous data, with mean OS ranging from one to 12 months. The main causes of death are lymphoma and associated infections [9,22,37, 38, 39, 40, 41, 42] (Table 4)**.** The main contributing factors to the unfavorable outcomes of L-HLH are late diagnosis and, consequently, a delay in providing adequate therapy, in addition to the heterogeneity of approaches resulting from the lack of randomized studies [22,38,39,42, 43, 44].

**Table 4:** Retrospective studies including lymphoma-associated hemophagocytic lymphohistiocytosis patients and overall survival outcomes.

**Study**	**Population**	**Outcome**
Han AR, et al. 2007	Retrospective, single-center, Korea *n* = 52 HLH 29 L-HLH 83 % T-cell lymphomas	Median OS = 36 days
Li F, et el. 2014	Retrospective, single-center, China *n* = 69 HLH 16 L-HLH, 15 T/NK-cell lymphomas	Median OS = 37 days
Yu JT, et el. 2013	Retrospective, single-center, China *n* = 30 L-HLH 69 % T-cell lymphomas	Median OS B-cell = 11 months Median OS T-cell = 3 months T-cell usually refractory to first line chemotherapy
Wei L, et al. 2020	Retrospective, single-center, China *n* = 43 All ENKTL	OS 34.4 % at 6 months All received etoposide (Etoposide) and/or dexamethasone-based treatment
Jin Z, et al. 2020	*n* = 8 All Hodgkin lymphomas	12-month OS = 56.3 %
Li B, et al. 2020	Retrospective, two centers in China *n* = 31 All B-cell NHL	Median OS = 1.5 months

OS: overall survival; ENKTL: extranodal natural-killer T-cell lymphoma; NHL: non-Hodgkin lymphoma.

Moreover, the subtype of lymphoma seems to be a relevant prognostic factor. Even in the context of L-HLH, B-cell lymphomas are usually associated with a better prognosis than NK/T-Cell lymphomas. In a multicenter retrospective study conducted in Japan with 132 cases of M-HLH, 108 patients had L-HLH, with 48.2 % and 12.2 % five-year OS for B-cell lymphoma and NK/T-cell lymphoma, respectively [44]. In another study, Wang et al. assessed the role of chemotherapy with a dose-adjusted etoposide (Etoposide) phosphate, prednisone, vincristine sulfate (Oncovin), cyclophosphamide, and doxorubicin hydrochloride (hydroxydaunorubicin) (DA-EPOCH) regimen in 55 patients with B-cell non-Hodgkin lymphoma (B-NHL), most of which were diffuse large B-cell lymphoma, and different subtypes of T-cell-NHL. The patients with B-NHL were more tolerant to treatment and received more treatment cycles, with five-year OS reaching 73 %. Conversely, patients with T-cell-NHL were less tolerant to treatment and responded less well to treatment, with only 3 % of patients surviving after 12 months [45]. Whether this difference derives from the increased clinical aggressiveness of NK/T-cell lymphoma or differences in the therapeutic approach to these subtypes, particularly the availability of anti-CD20 monoclonal antibodies for B-NHL, remains unclear.

Historically, the treatment of choice for HLH is based on the HLH-94 protocol, initially designed for children, but which has since proved feasible in adults, albeit more toxic and with lower response rates [37]. The main concept of this protocol is the weekly administration of etoposide (Etoposide) combined with dexamethasone, for its high potential for immunosuppression and, particularly, macrophage activity suppression, and the subsequent addition of cyclosporine (Cyclosporine capsules). A 6.2-year follow-up of the original study showed 54 % OS, which was higher for children undergoing consolidative allogeneic bone marrow transplantation (BMT) [46].

An updated HLH-2004 protocol has been proposed to improve response rates to HLH-94. The updated version primarily differed in bringing forward cyclosporine (Cyclosporine capsules) administration to the first few weeks of treatment. However, there was no additional clinical benefit. In fact, the strategy proved to be more toxic than the original protocol [47].

Although the HLH-94 protocol has not been validated for adult patients with L-HLH, some modified versions have been used. These modified versions consider the crucial role of etoposide (Etoposide) in depleting T-cell lymphocytes and suppressing immune hyperactivation, in addition to its anti-lymphoma effect [17,19,24]. Early etoposide (Etoposide) use combined with dexamethasone for immunomodulation in L-HLH leads to rapid control of hyperinflammation, an improved clinical condition, and reduced risk of permanent organ damage [15,48]. A Chinese retrospective study evaluated 66 patients with L-HLH divided into two groups. The first group included patients who had been treated with etoposide (Etoposide)-based protocols, whereas the second group included patients who had not been treated with the drug. The results showed a significant difference in response rate (73.1 % versus 42.9 %; p-value = 0.033) and median OS (25.8 months versus 7.8 months; p-value = 0.048) [49]. Bigenwald et al. analyzed a cohort of 71 patients with L-HLH and observed that treatment with etoposide (Etoposide) was independently associated with improved prognosis [50].

**Figure 2:**
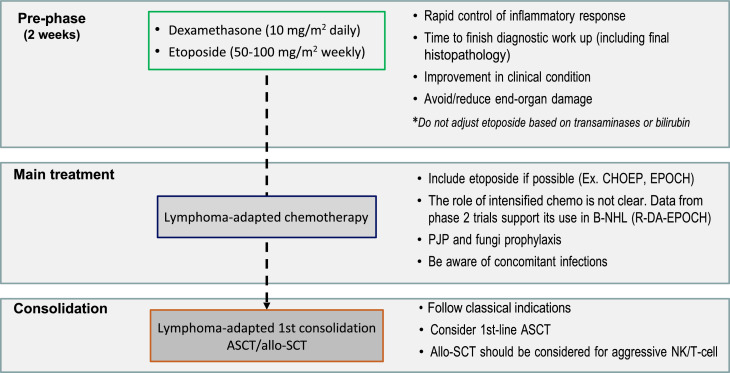
A suggested treatment approach for lymphoma-associated hemophagocytic lymphohistiocytosis. The two-stage approach aims to initially control hyperinflammation and T-cell proliferation based on weekly etoposide (Etoposide) and dexamethasone, followed by lymphoma treatment. CHOEP: Cyclophosphamide, Hydroxydaunorubicin, Oncovin, etoposide (Etoposide), Prednisone; EPOCH: etoposide (Etoposide), Prednisone, Oncovin (Vincristine), Cyclophosphamide, and Hydroxydaunorubicin (Doxorubicin); B-NHL: B-cell non-Hodgkin lymphoma; R-DA-EPOCH: Rituximab (Dose-Adjusted), etoposide (Etoposide), Prednisone, Oncovin (Vincristine), Cyclophosphamide, Hydroxydaunorubicin (Doxorubicin); ASCT: autologous stem-cell transplantation; PJP: *Pneumocystis jirovecii* pneumonia; Allo-SCT: allogenic stem-cell transplantation; NK: Natural killer cells.

The aggressive presentation of L-HLH is often associated with rapid clinical deterioration and significant laboratory changes. These changes delay lymphoma-specific treatment. Considering such factors, the MD Anderson Cancer Center published guidelines suggesting a two-stage approach, which has been widely adopted. The initial phase aims to control hyperinflammation and T-cell proliferation based on weekly etoposide (Etoposide) use in reduced doses (50–100 mg/m^2^) combined with corticosteroids. In the absence of an initial response, treatment is intensified with the liposomal doxorubicin, etoposide (Etoposide), and methylprednisolone (DEP) protocol. The second phase aims to treat the lymphoma as soon as clinical improvement and organ dysfunction reach permissive levels, notably lower ferritin, transaminases, and fibrinogen levels. Regimens with etoposide (Etoposide), such as EPOCH or DA-EPOCH, are recommended, along with rituximab in cases of B-NHL [15,19] (Figure 2). However, the use of high-dose regimens proved more toxic, with no additional benefit in NK/T-cell lymphomas [45].

Despite being included in the original HLH-94 protocol, the role of HSCT in adult HLH remains controversial and is reserved for cases of disease refractory to first-line therapy. In L-HLH, some studies suggest that autologous HSCT is beneficial as a first-line consolidation therapy, but data are scarce, thus preventing the generalization of this approach. Consolidation should follow the usual indications in lymphoma treatment and be discussed individually in severe cases [45,51]. Allogeneic HSCT should be considered for first-line candidates with NK/T-NHL, given the poor prognosis of this cohort, and in cases refractory to induction treatment, particularly in reduced-intensity conditioning (RIC) [12,15,16].

Considering that exacerbated cytokine production in HLH plays a key role in the Janus Kinase-Signal Transducer and Activator of Transcription (JAK-STAT) pathway [52], ruxolitinib, a JAK 1 and 2 inhibitor, has been tested as a potential targeted treatment, with promising results. Single-arm studies and some case series demonstrate that this drug is effective in secondary HLH with different associated triggers in first-line settings and refractory cases [53, 54, 55]. The most representative cohort is found in a Chinese study on 70 patients with L-HLH, 36 of whom were treated with the ruxolitinib, liposomal doxorubicin, etoposide (Etoposide), and dexamethasone (R-DED) protocol and 34 with etoposide (Etoposide) combined with dexamethasone, followed by lymphoma-specific treatment in both arms. Patients in the R-DED group had \ higher overall response rate (83.3 % versus 54.8 %; p-value = 0.011) and median OS (5 months versus 1.5 months; p-value = 0.003). Moreover, this cohort was mainly composed of patients with T-cell lymphoma (78.6 %), generally associated with a more reserved prognosis when associated with HLH [56].

The effect of anti-cytokine therapies on L-HLH is still unclear. The interleukin-1 receptor agonist anakinra has been the most frequently used in HLH associated with rheumatologic diseases (often termed macrophage activation syndrome). However, its efficacy in the context of malignancy is questionable [57,58]. Interleukin-6 blockade with tocilizumab has been extrapolated from its use in cytokine release syndrome and coronavirus disease 2019, but data on HLH are limited, with a recent series showing increased mortality from infections when tocilizumab was used in the context of M-HLH [59].

HLH triggered by lymphoma treatment should be subjected to differential diagnosis for L-HLH. This condition has been more frequently observed with the development and increasingly widespread use of new therapies, such as checkpoint inhibitors, CAR T-cells, and bispecific antibodies. Mild-to-moderate cytokine release syndrome is expected because of these therapies, with good response to tocilizumab and anakinra [60]. More severe cases that progress with HLH characteristics are less frequent and generally managed using anti-interleukin drugs, however there is the lack of robust data in the literature [61]. For other cases, first-line anti-interleukin therapy in L-HLH is not routinely recommended until more robust evidence demonstrates efficacy and safety.

Despite available therapies, most patients with L-HLH, particularly those with NK/T-cell lymphoma, fail to respond to first-line therapy or relapse after a brief response. Relapsed or refractory disease is associated with high mortality, usually due to the progression of HLH/lymphoma or secondary infections. No standard treatment is available, and, as a rule, the approach should be individualized, focusing on controlling the neoplasia and other concomitant predisposing conditions.

High-dose chemotherapy was evaluated using the DEP protocol in 63 patients, 29 of whom had L-HLH. The overall response rate was 76.2%, reaching 75.7% in L-HLH. The reported median OS was 28 weeks [62]. Other therapeutic options studied so far include ruxolitinib, alemtuzumab, and emapalumab, an anti-interferon gamma, albeit with little data on L-HLH [16,52,63]. A recent study investigated the use of emapalumab in patients with hematologic M-HLH, the majority of whom had L-HLH and had undergone extensive prior treatment. Emapalumab did not yield promising results, showing limited clinical efficacy in this population, although a few patients demonstrated improvements in HLH-related biomarkers [64].

Regardless of the therapeutic approach, treatments for refractory L-HLH are more toxic and less likely to lead to long-lasting remission. Therefore, allogeneic BMT should be considered for candidates with an available donor, preferably using reduced intensity conditioning (RIC). Studies have shown that this approach reaches 50–75 % OS and that the best results are found in combination with alemtuzumab prior to RIC [65,66].

High clinical suspicion, prompt immunosuppressive therapy, and adequate clinical support remain the pillars of successful L-HLH treatment. An improved understanding of the pathophysiology of this condition, elucidation of molecular pathways, and genetic alterations that may contribute to the exacerbated inflammatory response have spurred the development of individualized protocols and the exploration of new agents as adjuvants. Future collaborative studies are crucial for assessing the best therapeutic strategies, particularly in patients with HLH associated with NK/T-cell lymphoma, which has a poor prognosis.

## Conflicts of interest

none
